# Associations between screen time and lower psychological well-being among children and adolescents: Evidence from a population-based study

**DOI:** 10.1016/j.pmedr.2018.10.003

**Published:** 2018-10-18

**Authors:** Jean M. Twenge, W. Keith Campbell

**Affiliations:** a San Diego State University, United States of America; b University of Georgia, United States of America

## Abstract

Previous research on associations between screen time and psychological well-being among children and adolescents has been conflicting, leading some researchers to question the limits on screen time suggested by physician organizations. We examined a large (*n* = 40,337) national random sample of 2- to 17-year-old children and adolescents in the U.S. in 2016 that included comprehensive measures of screen time (including cell phones, computers, electronic devices, electronic games, and TV) and an array of psychological well-being measures. After 1 h/day of use, more hours of daily screen time were associated with lower psychological well-being, including less curiosity, lower self-control, more distractibility, more difficulty making friends, less emotional stability, being more difficult to care for, and inability to finish tasks. Among 14- to 17-year-olds, high users of screens (7+ h/day vs. low users of 1 h/day) were more than twice as likely to ever have been diagnosed with depression (RR 2.39, 95% CI 1.54, 3.70), ever diagnosed with anxiety (RR 2.26, CI 1.59, 3.22), treated by a mental health professional (RR 2.22, CI 1.62, 3.03) or have taken medication for a psychological or behavioral issue (RR 2.99, CI 1.94, 4.62) in the last 12 months. Moderate use of screens (4 h/day) was also associated with lower psychological well-being. Non-users and low users of screens generally did not differ in well-being. Associations between screen time and lower psychological well-being were larger among adolescents than younger children.

## Highlights

• More hours of screen time are associated with lower well-being in ages 2 to 17.
• High users show less curiosity, self-control, and emotional stability.
• Twice as many high (vs. low) users of screens had an anxiety or depression diagnosis.
• Non-users and low users did not differ in well-being.
• Associations with well-being were larger for adolescents than for children.

## 1 Introduction

A growing proportion of children and adolescents' leisure time is spent with screens including smartphones, tablets, gaming consoles, and televisions (Common Sense Media, 2015; Twenge et al., 2019), raising concerns about the effect of screen time on well-being among parents, health professionals, and educators (e.g., Kardaras, 2017). These concerns have prompted physician organizations such as the American Academy of Pediatrics (AAP) to recommend that parents limit children's daily screen time, with specific time limits for preschool children and a general suggestion of limiting time on screens for older children and adolescents (Radesky and Christakis, 2016). In addition, the World Health Organization recently decided to include gaming disorder in the 11th revision of the International Classification of Diseases (WHO, 2018).

Associations between screen time and poor health outcomes such as obesity and lack of exercise have been well-documented (e.g., Chiasson et al., 2016; de Jong et al., 2013; Dumuid et al., 2017; Poitras et al., 2017). However, research exploring associations between screen time and more psychological aspects of well-being among children and adolescents has been inconsistent. Some studies find significant associations between screen time and low well-being (Babic et al., 2017; Page et al., 2010; Romer et al., 2013; Rosen et al., 2014; Twenge et al., 2018a, Twenge et al., 2018b; Yang et al., 2013), while others find null effects or even benefits with greater screen time (Granic et al., 2014; Odgers, 2018; Przybylski and Weinstein, 2018; Valkenburg and Peter, 2009). Thus, some have suggested that more research is needed before concluding that screen time limits are justified, arguing that valuable physician appointment time should not be devoted to discussing screen time without sufficient evidence for significant associations with well-being (Przybylski and Weinstein, 2017, Przybylski and Weinstein, 2018). Some researchers have made similar statements about the WHO characterizing gaming disorder as a mental health issue, maintaining that associations between gaming and psychological well-being are not substantial or consistent enough to justify such a classification (Davis, 2018; van Rooij et al., 2018).

Theories and research on psychological well-being support the notion of a broad concept including emotional stability, positive interpersonal relationships, self-control, and indicators of flourishing (Diener et al., 1999; Ryff, 1995) as well as diagnoses of mood disorders such as anxiety or depression (Manderscheid et al., 2010). Low emotional stability, disrupted relationships, and low self-control have all been implicated in greater morbidity and mortality (Graham et al., 2017; Shipley et al., 2007; Shor et al., 2013; Turiano et al., 2015), and mental health issues such as mood disorders are a significant risk factor for morbidity and mortality, including via non-suicidal self-harm behaviors, suicide attempts, and completed suicides (Hawton et al., 2013; Murray et al., 2012).

In terms of prevention, establishing possible causes and outcomes of low psychological well-being is especially important for child and adolescent populations. Half of mental health problems develop by adolescence (Erskine et al., 2015). Thus, there is an acute need to identify factors linked to mental health issues that are amenable to intervention in this population, as most antecedents (e.g., genetic predisposition, trauma, poverty) are difficult or impossible to influence. Compared to these more intractable antecedents of mental health, how children and adolescents spend their leisure time is more amenable to change.

To our knowledge, few if any previous studies have examined a broad array of psychological well-being items in relation to screen time. Moreover, although other studies have examined associations between screen time and *symptoms* of anxiety and depression, no previous study we know of has examined associations between screen time and actual *diagnoses* of anxiety or depression or reports of professional treatment for mental health issues. Furthermore, it is crucial for measures of screen time to include not just television but more recently introduced digital media including electronic gaming, smartphones, tablets, and computers. In addition, studies using the same items to assess several age groups of children and adolescents are rare, which is unfortunate as age may be a significant moderator of associations between screen time and psychological well-being.

The current research aims to examine associations between screen time and a diverse array of measures of psychological well-being (including emotional stability, relationships with caregivers, self-control, diagnoses of mood disorders, and treatment of mental health issues) among a large population-based survey of the caregivers of children and adolescents ages 2 to 17 collected in 2016 in the U.S.

## 2 Method

### 2.1 Participants

Participants were the caregivers of 44,734 children and adolescents 2 years of age and older in the U.S. in the National Survey of Children's Health (NSCH) conducted in 2016 by the U.S. Census Bureau. As many items on psychological well-being were asked only of caregivers with children ages 2 and over, we restricted our analyses to children ages 2 to 17.

Households were contacted by mail at random to identify those with children or adolescents 17 years old or younger. In every household, one child was randomly selected to be the subject of the survey. The survey was administered either online or on paper, with an oversampling of children with special health care needs. The response rate was 40.7%. Data are publicly available on the NSCH website.

We excluded children and adolescents with at least one of 8 major conditions that might affect their day-to-day functioning: Autism, blindness, cerebral palsy, deafness, Down Syndrome, developmental delay, epilepsy, or intellectual disability (mental retardation), as these were related to both well-being and to screen time. For example, among 14- to 17-year-olds, 33% of those with zero daily use of screens had one of these conditions, compared to an overall 10.1% base rate. The survey's comprehensive screener for special health care needs could not be used as it also includes those who receive any mental health care services, which is a variable of interest. These exclusions resulted in a sample *n* of 40,337.

In the final sample, the children and adolescents were 49.8% male and 50.2% female and were 71% White, 16% Hispanic, 6% Black, and 7% other. Family income was widely distributed, with 9% of children below the 100% poverty level and 44% with family incomes at the 400% level or more. The sample was designed to be nationally representative of all U.S. children at these ages but underrepresents some groups due to lower response rates.

We grouped children and adolescents into four categories based on age that roughly correspond to educational levels: Preschoolers 2 to 5 years old (*n* = 9361), elementary schoolers 6 to 10 years old (*n* = 10,668), middle schoolers 11 to 13 years old (*n* = 7555), and high schoolers 14 to 17 years old (*n* = 12,753). These categories also correspond to the structure of the survey, with some questions only asked of the caregivers of preschool children and others asked only of children 6 years of age and up.

### 2.2 Measures

The survey asked two items about screen time. First, “On an average weekday, about how much time does [child's name] spend in front of a TV watching TV programs, videos, or playing video games?” Second, “On an average weekday, about how much time does [child's name] spend with computers, cell phones, handheld video games, and other electronic devices, doing things other than schoolwork?” For both, response choices were recoded to none = 0, less than an hour = 0.5, an hour = 1, 2 h = 2, 3 h = 3, and 4 or more hours = 5. For means, see Table 1.

**Table 1:** Approximate hours a day of screen use by age group, U.S., 2016.

	2 to 5	6 to 10	11 to 13	14 to 17	*d*
TV and video games	1.46 (1.09)	1.53 (1.10)	1.80 (1.39)	1.89 (1.39)	0.34
Electronic devices	0.82 (0.96)	1.25 (1.11)	2.00 (1.40)	2.70 (1.53)	1.46
Total screen time	2.28 (1.72)	2.78 (1.95)	3.80 (2.36)	4.59 (2.50)	1.06

Note: 1. SDs in parentheses.

We added together the estimated number of hours spent on TV/video games and on digital media devices to create a measure of total screen time and recoded the results into 8 categories: None (no screen time), <1 h (0.01 to 0.99), 1 h (1.00 to 1.49), 2 h (1.50 to 2.49), 3 h (2.50 to 3.49), 4 h (3.50 to 4.49), 5 h (4.50 to 5.49), 6 h (5.50 to 6.49) and 7 h or more (6.50 and higher). Among the two older groups, very few reported no screen time at all (*n* = 46 for 11- to 13-year-olds and *n* = 24 for 14- to 17-year-olds), so these cells should be interpreted with caution.

We examined all items in the NSCH survey that measured psychological well-being, broadly construed (see Supplemental material for item wording including response choices). Most items did not correlate highly enough to be combined into scales and are thus analyzed as single items. The exceptions were three items measuring how easy the child is and four items measuring self-control. We coded all items so that higher scores indicated higher well-being.

### 2.3 Analysis plan

Analyses included controls for possible confounding variables: child race (dummy variables for Black, Hispanic, and Other, with non-Hispanic White as the comparison group), child sex, child age, household adults' highest grade completed (continuous, using the detailed item including college education), family poverty ratio (a measure of family income), and family structure (living with two biological/adoptive parents vs. not). We did not weight analyses and did not replace missing data.

For items on a continuum, we report means in tables and percent low in well-being in figures; categorical items (e.g., yes or no, such as diagnoses of anxiety or depression) are reported as percentages in both. The tables report effect sizes (*d*, or difference in terms of standard deviations) as well as *p*-values for *t*-tests comparing means at different levels of use. The text reports relative risk (RR) with 95% confidence intervals (CIs) for dichotomized items.

We first examine items asked of caregivers of several ages of children and then those asked only of caregivers of preschool children. Given the curvilinear relationship between screen time and well-being found in previous research (Przybylski and Weinstein, 2017; Twenge et al., 2018b), we identified the inflection point at which the trend in well-being moved from positive to negative to inform our analyses (Simonsohn, 2017). Thus, we compare no use to low levels of use, low use to moderate use, and low use to high use.

## 3 Results

### 3.1 Age differences in screen time

**Fig. 1:**
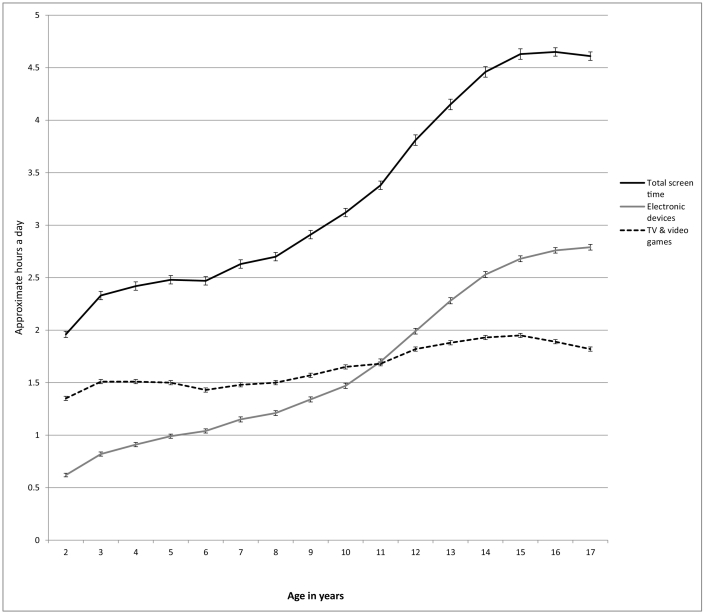
Hours per day spent on all screens, electronic devices, and TV and video games by individual years of age, U.S., 2016. Error bars are ±1 SE.

Total screen time averaged 3.20 h a day (SD = 2.40) and was progressively higher among older children, primarily driven by more time spent on electronic devices (see Table 1 and Fig. 1). The largest increase in screen time occurred between elementary school and middle school. By high school (ages 14 to 17), adolescents spent 4 h and 35 min a day with screens according to caregivers' reports.

### 3.2 Screen time and well-being

**Fig. 2:**
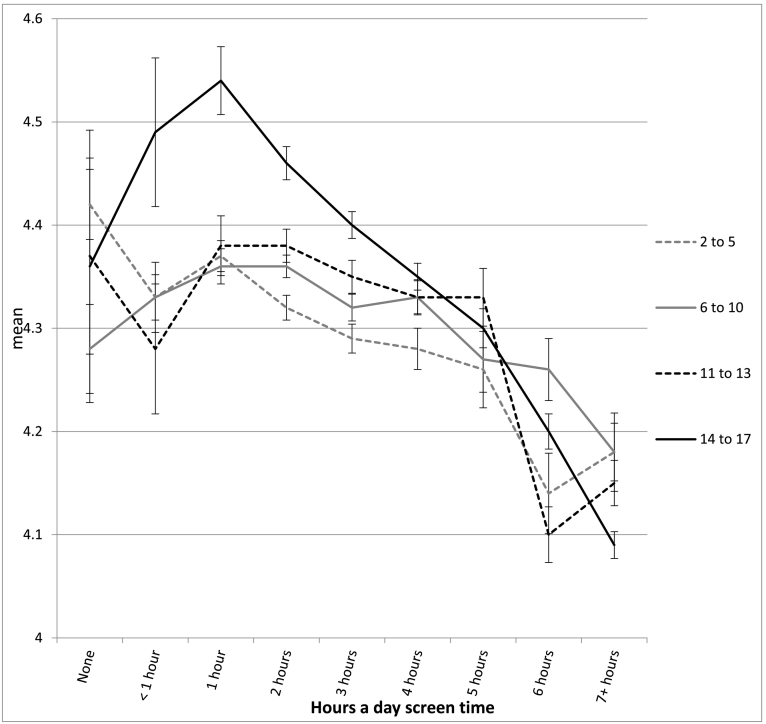
Mean score on the easy child index (1–5), by age and level of screen time, with controls, U.S., 2016. Error bars are ±1 SE.

**Fig. 3:**
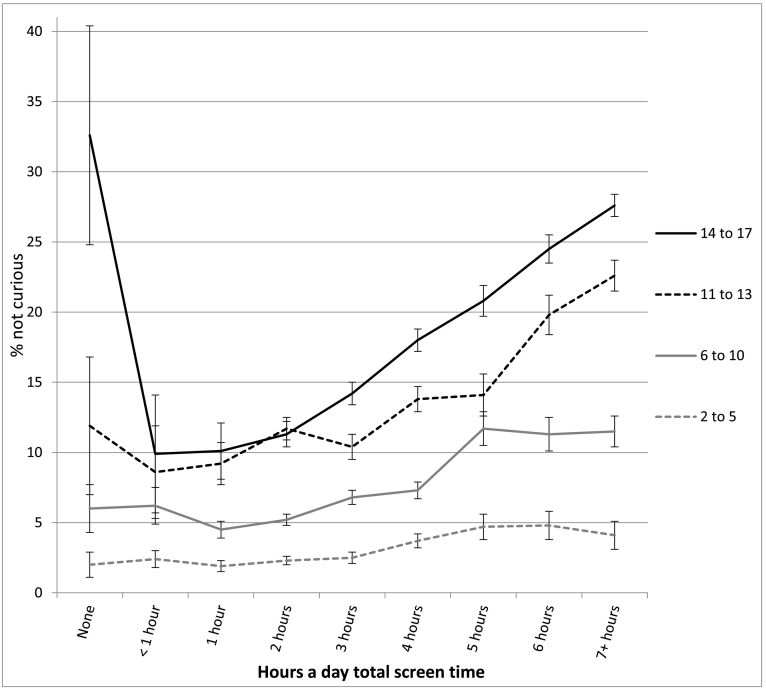
Percentage not curious or interested in learning new things, by age and level of screen time, with controls, U.S., 2016. Error bars are ±1 SE.

**Fig. 4:**
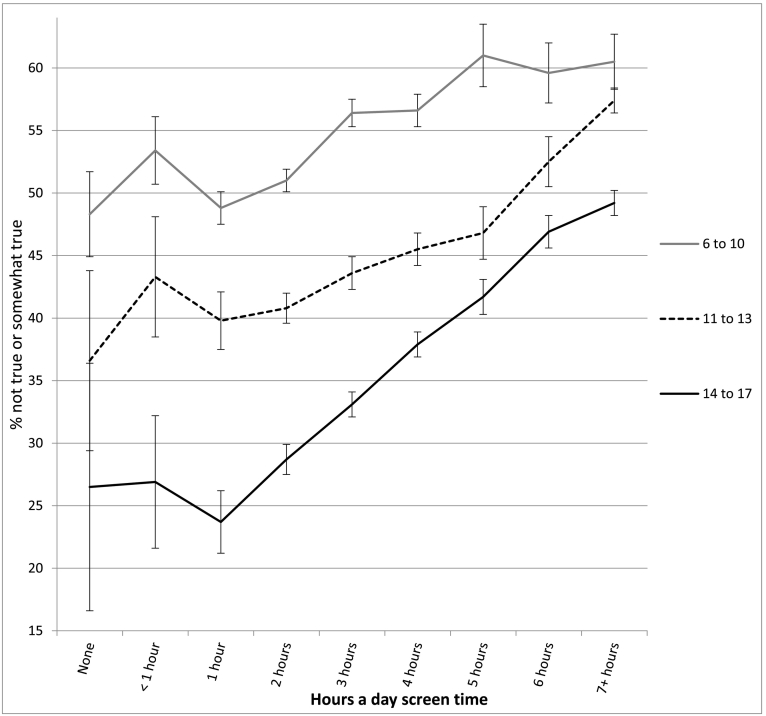
Percentage who do not stay calm when challenged, by age and level of screen time, with controls, U.S., 2016. Error bars are ±1 SE.

**Fig. 5:**
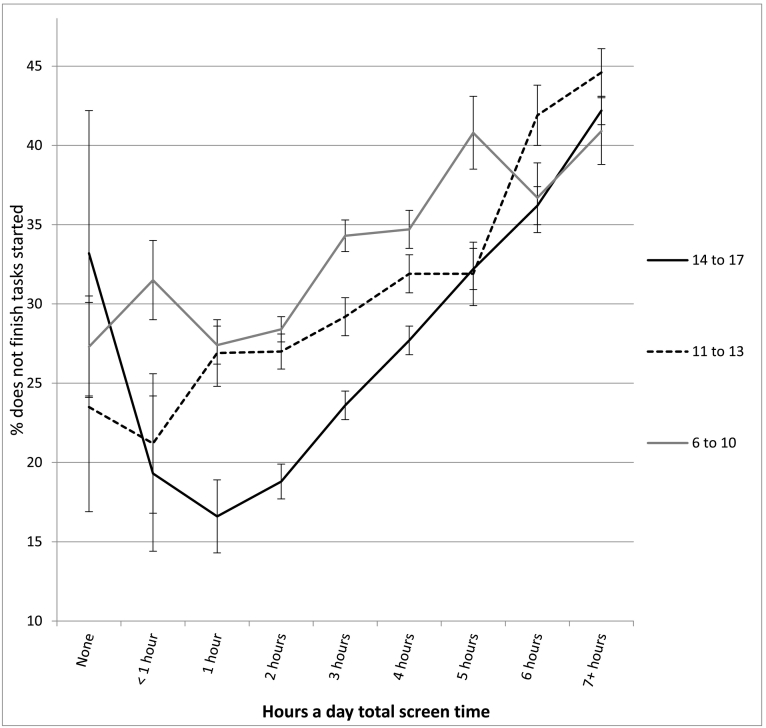
Percentage who do not finish tasks started, by age and level of screen time, with controls, U.S., 2016. Error bars are ±1 SE.

**Fig. 6:**
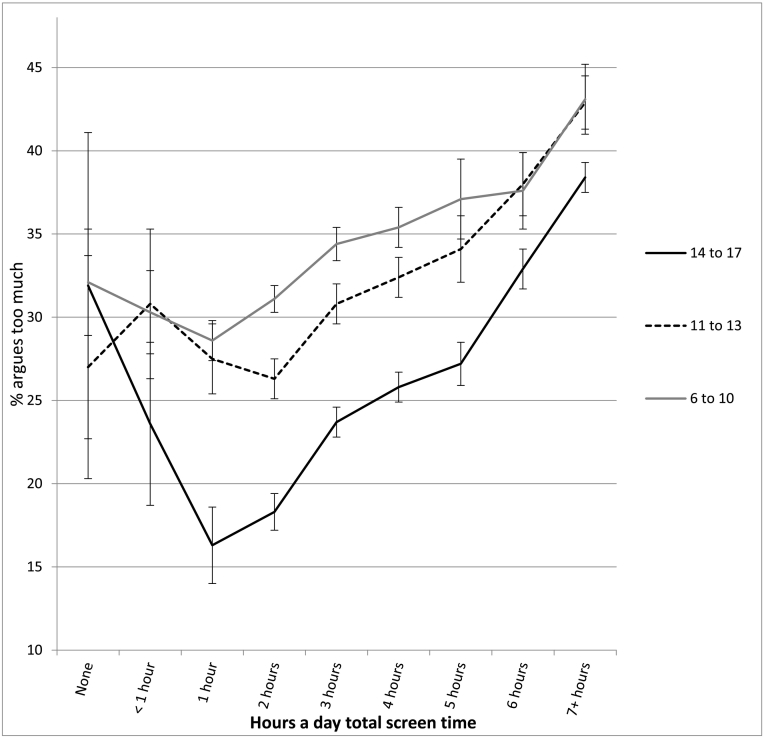
Percentage who argue too much with their caregivers, by age and level of screen time, with controls, U.S., 2016. Error bars are ±1 SE.

The association between screen time and well-being was not linear and showed an inflection point at 1 h/day of use for most measures (see Table 2 and Fig. 2, Fig. 3, Fig. 4, Fig. 5, Fig. 6). With one exception (the item on curiosity), well-being did not differ significantly between those spending no time on screens and those spending an hour a day. After an hour a day, however, increasing screen time was generally linked to progressively lower psychological well-being. In terms of relative risk (RR), high users of screens (7+ h/day) carried twice the risk of low well-being as low users (1 h/day), including not staying calm (e.g., among 14- to 17-year-olds, RR 2.08, 95% CI 1.72, 2.50), not finishing tasks (RR 2.53, CI 2.01, 3.20), not being curious (RR 2.72, CI 2.00, 3.71), and arguing too much with caregivers (RR 2.34, CI 1.85, 2.97; see Fig. 2, Fig. 3, Fig. 4, Fig. 5, Fig. 6). High (vs. low) users of screens were also described as more difficult to care for. Effect sizes were generally larger among adolescents than among children.

**Table 2:** Well-being means by hours per day of total screen time (with controls) among age groups and *d*'s comparing cells, U.S., 2016.

	None (0)	<1 h	1 h	2 h	3 h	4 h	5 h	6 h	7+ h	*d* 0 vs. 1 h	*d* 1 h vs. 4 h	*d* 1 h vs. 7+ h
Easy child (3 items)												
2 to 5 (preschool)	4.42 (0.53) 320	4.33 (0.61) 749	4.37 (0.57) 1707	4.32 (0.61) 2687	4.29 (0.62) 1843	4.28 (0.63) 912	4.26 (0.66) 280	4.14 (0.73) 243	4.18 (0.67) 263	−0.09	−0.15*	−0.33*
6 to 10 (elem.)	4.28 (0.65) 215	4.33 (0.63) 348	4.36 (0.63) 1457	4.36 (0.62) 3203	4.32 (0.63) 2187	4.33 (0.64) 1483	4.27 (0.65) 397	4.26 (0.63) 431	4.18 (0.69) 496	0.13	−0.05	−0.28*
11 to 13 (middle)	4.37 (0.65) 46	4.28 (0.72) 104	4.38 (0.66) 477	4.38 (0.60) 1621	4.35 (0.62) 1537	4.33 (0.63) 1464	4.33 (0.60) 525	4.21 (0.70) 566	4.15 (0.73) 895	0.02	−0.08	−0.33*
14 to 17 (h.s.)	4.36 (0.99) 24	4.49 (0.58) 80	4.54 (0.51) 370	4.46 (0.57) 1679	4.40 (0.57) 2488	4.35 (0.60) 2468	4.30 (0.66) 1118	4.20 (0.71) 1370	4.09 (0.77) 2547	0.33	−0.32*	−0.61*

Curious												
2 to 5 (preschool)	2.98 (0.12) 319	2.98 (0.14) 752	2.98 (0.12) 1716	2.98 (0.15) 2705	2.98 (0.16) 1853	2.96 (0.21) 919	2.96 (0.22) 280	2.95 (0.25) 245	2.96 (0.22) 266	0.00	−0.13*	−0.15
6 to 10 (elem.)	2.94 (0.22) 215	2.94 (0.23) 349	2.95 (0.19) 1462	2.95 (0.22) 3225	2.93 (0.26) 2200	2.93 (0.29) 1488	2.88 (0.34) 401	2.88 (0.35) 434	2.88 (0.37) 504	0.05	−0.08*	−0.30*
11 to 13 (middle)	2.88 (0.32) 47	2.91 (0.25) 105	2.91 (0.28) 480	2.88 (0.32) 1631	2.89 (0.30) 1537	2.86 (0.36) 1474	2.86 (0.36) 533	2.79 (0.46) 571	2.76 (0.47) 901	0.11	−0.15*	−0.37*
14 to 17 (h.s.)	2.64 (0.58) 24	2.90 (0.26) 82	2.90 (0.30) 375	2.88 (0.31) 1691	2.86 (0.34) 2501	2.82 (0.39) 2485	2.79 (0.43) 1132	2.74 (0.48) 1371	2.71 (0.49) 2583	0.78*	−0.21*	−0.41*

No difficulty making friends												
2 to 5 (preschool)	2.87 (0.35) 181	2.89 (0.31) 400	2.90 (0.30) 1112	2.91 (0.29) 1980	2.90 (0.32) 1405	2.86 (0.38) 707	2.81 (0.41) 204	2.85 (0.38) 191	2.84 (0.39) 209	0.10	−0.12*	−0.19*
6 to 10 (elem.)	2.81 (0.42) 211	2.83 (0.40) 346	2.85 (0.38) 1454	2.85 (0.38) 3189	2.83 (0.41) 2176	2.82 (0.43) 1470	2.78 (0.48) 396	2.77 (0.45) 429	2.77 (0.47) 492	0.10	−0.07*	−0.20*
11 to 13 (middle)	2.74 (0.53) 47	2.80 (0.45) 104	2.78 (0.47) 473	2.82 (0.43) 1620	2.78 (0.48) 1525	2.79 (0.44) 1464	2.82 (0.43) 522	2.70 (0.52) 569	2.66 (0.58) 888	0.08	0.02	−0.22*
14 to 17 (h.s.)	2.81 (0.49) 23	2.76 (0.48) 81	2.88 (0.36) 367	2.85 (0.40) 1678	2.82 (0.42) 2476	2.79 (0.46) 2464	2.76 (0.49) 1113	2.72 (0.51) 1355	2.66 (0.58) 2521	0.19	−0.20*	−0.40*

Calm when challenged												
6 to 10 (elem.)	2.46 (0.60) 211	2.42 (0.58) 344	2.48 (0.54) 1450	2.45 (0.56) 3190	2.39 (0.57) 2176	2.37 (0.60) 1465	2.32 (0.60) 394	2.35 (0.59) 428	2.32 (0.62) 491	0.04	−0.19*	−0.29*
11 to 13 (middle)	2.62 (0.54) 47	2.54 (0.54) 104	2.56 (0.56) 475	2.57 (0.53) 1619	2.53 (0.56) 1521	2.51 (0.56) 1459	2.51 (0.56) 524	2.43 (0.60) 567	2.35 (0.62) 887	−0.11	0.09	−0.35*
14 to 17 (h.s.)	2.70 (0.58) 23	2.68 (0.56) 81	2.75 (0.45) 367	2.70 (0.49) 1677	2.66 (0.50) 2472	2.60 (0.54) 2462	2.55 (0.57) 1110	2.48 (0.60) 1352	2.45 (0.62) 2523	0.11	−0.29*	−0.50*

Works to finish tasks started												
6 to 10 (elem.)	2.71 (0.48) 211	2.66 (0.49) 345	2.72 (0.46) 1450	2.70 (0.48) 3182	2.65 (0.50) 2175	2.64 (0.52) 1465	2.58 (0.55) 392	2.61 (0.55) 430	2.57 (0.56) 491	0.02	−0.16*	−0.31*
11 to 13 (middle)	2.75 (0.50) 47	2.79 (0.39) 104	2.72 (0.46) 474	2.72 (0.46) 1625	2.70 (0.47) 1522	2.67 (0.50) 1461	2.67 (0.50) 525	2.55 (0.57) 566	2.51 (0.59) 887	0.06	−0.10*	−0.39*
14 to 17 (h.s.)	2.67 (0.49) 24	2.78 (0.45) 81	2.83 (0.37) 366	2.81 (0.39) 1675	2.76 (0.43) 2468	2.71 (0.47) 2455	2.66 (0.52) 1114	2.60 (0.57) 1352	2.54 (0.58) 2523	0.38	−0.26*	−0.52*

Does not argue too much												
6 to 10 (elem.)	2.66 (0.51) 209	2.64 (0.57) 346	2.67 (0.55) 1452	2.64 (0.58) 3193	2.60 (0.60) 2178	2.58 (0.61) 1467	2.56 (0.63) 393	2.58 (0.59) 430	2.48 (0.67) 490	0.04	0.16*	−0.33*
11 to 13 (middle)	2.69 (0.56) 47	2.54 (0.55) 104	2.68 (0.55) 476	2.69 (0.54) 1621	2.63 (0.58) 1524	2.62 (0.59) 1465	2.61 (0.59) 526	2.54 (0.65) 569	2.47 (0.68) 887	−0.02	−0.10*	−0.33*
14 to 17 (h.s.)	2.60 (0.66) 23	2.71 (0.55) 80	2.81 (0.46) 366	2.79 (0.46) 1681	2.73 (0.50) 2477	2.71 (0.53) 2461	2.68 (0.57) 1114	2.61 (0.60) 1354	2.52 (0.67) 2530	0.45	−0.19*	−0.45*

Ever diagnosed with anxiety												
11 to 13 (middle)	9.6% (0.29) 47	6.8% (0.25) 105	9.9% (0.30) 481	7.6% (0.26) 1634	10.0% (0.30) 1540	8.5% (0.28) 1477	9.3% (0.29) 532	11.2% (0.32) 573	12.2% (0.33) 904	0.01	0.05	0.07
14 to 17 (h.s.)	11.5% (0.32) 24	12.0% (0.33) 80	7.9% (0.26) 374	8.4% (0.28) 1698	9.7% (0.30) 2504	12.2% (0.33) 2489	13.4% (0.34) 1131	17.7% (0.38) 1374	18.1% (0.39) 2578	−0.13	0.13*	0.27*

Ever diagnosed with depression												
11 to 13 (middle)	4.6% (0.21) 47	1.6% (0.12) 105	3.7% (0.19) 481	1.9% (0.14) 1629	4.1% (0.19) 1543	3.8% (0.19) 1479	4.3% (0.21) 534	5.4% (0.23) 573	7.2% (0.26) 906	−0.05	0.05	0.15*
14 to 17 (h.s.)	10.2% (0.30) 24	8.3% (0.28) 82	5.3% (0.23) 376	5.1% (0.23) 1700	6.3% (0.24) 2508	8.6% (0.28) 2493	8.8% (0.29) 1131	11.6% (0.32) 1379	12.7% (0.33) 2582	−0.20	0.12*	0.23*

Treated or needed to be treated by mental health professional, past 12 months												
11 to 13 (middle)	7.6% (0.25) 47	10.4% (0.30) 104	13.5% (0.34) 480	10.5% (0.30) 1633	12.6% (0.33) 1539	12.2% (0.33) 1474	9.8% (0.30) 532	14.8% (0.36) 573	18.1% (0.39) 904	0.18	−0.04	0.12*
14 to 17 (h.s.)	25.8% (0.42) 24	15.9% (0.37) 82	9.8% (0.29) 374	11.5% (0.32) 1693	12.8% (0.34) 2496	14.1% (0.35) 2487	17.0% (0.38) 1128	20.7% (0.41) 1373	21.9% (0.41) 2578	−0.53	0.13*	0.30*

Took medication for psychological issue, past 12 months												
11 to 13 (middle)	9.0% (0.29) 46	6.2% (0.24) 105	8.6% (0.28) 479	6.9% (0.25) 1623	8.5% (0.28) 1529	9.4% (0.29) 1473	9.1% (0.29) 529	12.4% (0.33) 571	13.3% (0.34) 894	−0.01	0.03	0.15*
14 to 17 (h.s.)	11.7% (0.32) 23	11.7% (0.32) 82	5.5% (0.22) 372	8.2% (0.28) 1686	8.6% (0.28) 2492	9.9% (0.30) 2481	12.1% (0.33) 1116	14.9% (0.36) 1366	16.1% (0.37) 2562	−0.27	0.15*	0.30*

Notes: 1. Within levels of screen time, numbers in each cell are: well-being means, SDs in parentheses, and *n*'s. 2. *d* = effect size corresponding to difference in standard deviations. 3. * = *t*-test comparing cells significant at *p* < .05. 4. For diagnoses, treatment, and medication, base rates were high enough for reliable comparisons only among the two older age groups.

In most cases, moderate users of screens (4 h/day) were also significantly lower in well-being than low users (1 h/day), though with lower effect sizes (see Table 2). Among 14- to 17-year-olds, moderate users (vs. low users) were 78% more likely to not be curious (RR 1.78, CI 1.30, 2.43), 60% more likely to not stay calm when challenged (RR 1.60, CI 1.32, 1.93), 66% more likely to not finish tasks they started (RR 1.66, CI 1.31, 2.11), and 57% more likely to argue too much with their caregivers (RR 1.57, CI 1.24, 2.00; see Fig. 2, Fig. 3, Fig. 4, Fig. 5, Fig. 6). As with the comparisons between low and high use, differences in well-being between low and moderate users were smaller among younger children than among older adolescents.

### 3.3 Screen time and diagnoses of anxiety and depression

**Fig. 7:**
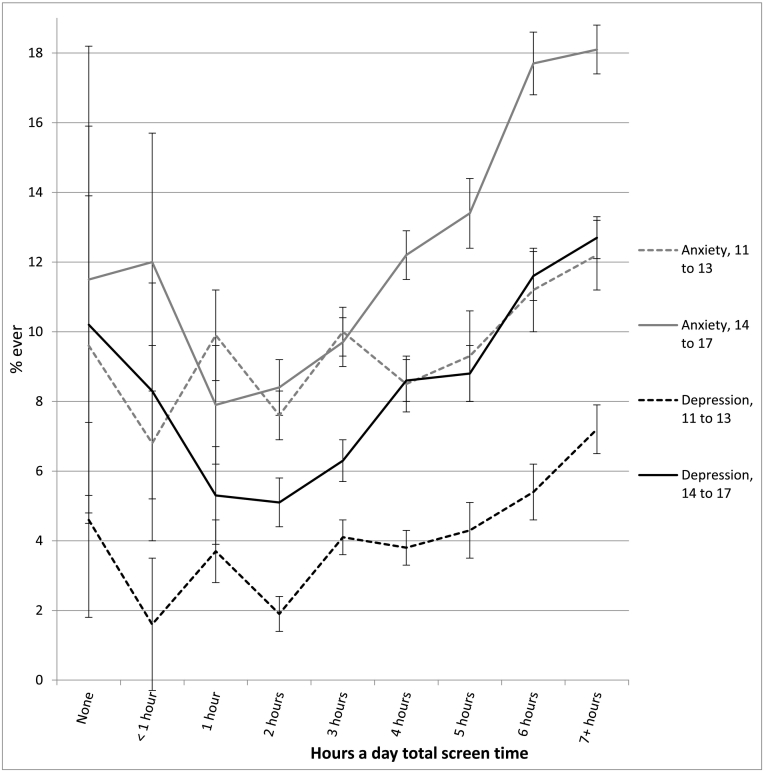
Percentage ever diagnosed with anxiety or depression, by age and level of screen time, with controls, U.S., 2016. Error bars are ±1 SE.

**Fig. 8:**
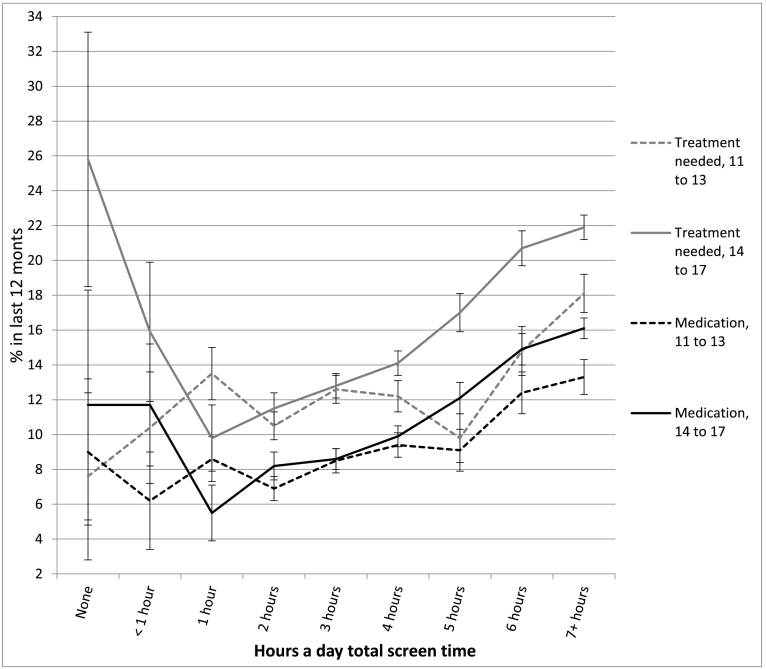
Percentage receiving mental health treatment and percentage taking medication for psychological issues during the last 12 months, by age and level of screen time, with controls, U.S., 2016. Error bars are ±1 SE.

High users of screens were also significantly more likely to have been diagnosed with anxiety or depression. Fourteen to 17-year-olds spending 7+ h/day with screens (vs. 1 h/day) were more than twice as likely to ever have been diagnosed with depression (RR 2.39, 95% CI 1.54, 3.70) or anxiety (RR 2.26, CI 1.59, 3.22; see Fig. 7). High users of screens were also more likely to have seen or needed to have been seen by a mental health professional (RR 2.22, CI 1.62, 3.03), and more likely to have taken medication for a psychological issue (RR 2.99, CI 1.94, 4.62; see Fig. 8) in the last 12 months. Moderate use was also linked to a greater risk of depression (RR 1.61, CI 1.03, 2.52) and anxiety diagnoses (RR 1.52, CI 1.06, 2.18) among 14- to 17-year-olds, though not among 11- to 13-year-olds.

### 3.4 Screen time and well-being items asked only of caregivers of preschool children

**Fig. 9:**
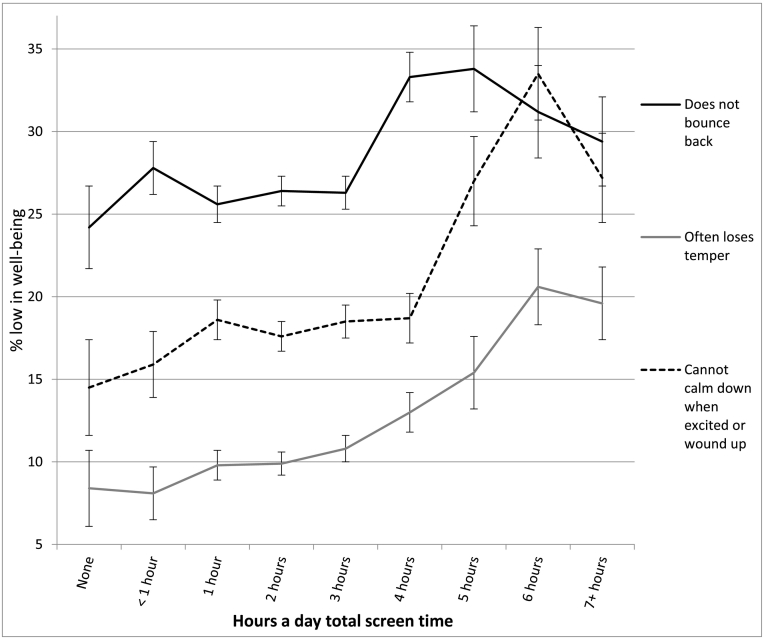
Percentage who do not bounce back, often lose their temper, or cannot calm down when excited or wound up, by total screen time, 2- to 5-year-old children, with controls, U.S., 2016. Error bars are ±1 SE.

We next examined the items asked only of caregivers of preschool children. High users of screens were more likely to lose their temper, less likely to calm down when excited, and less likely to switch tasks without anxiety or anger (see Table 3 and Fig. 9). The largest effect size appeared for self-control (*d* = −0.41), which included perseverance, sitting still, completing simple tasks, and not becoming distracted; both high and moderate users of screens displayed significantly lower self-control than low users. In terms of relative risk, high (vs. low) users of screens were twice as likely to often lose their temper (RR 1.99, CI 1.44, 2.77) and were 46% more likely to not be able to calm down when excited (RR 1.46, CI 1.13, 1.88). Preschool children with moderate screen use were also lower in well-being than those at low levels of use (see Table 3). In terms of relative risk, moderate users (vs. low users) were 30% more likely to not bounce back (RR 1.30, CI 1.15, 1.47) and 33% more likely to lose their temper (RR 1.33, CI 1.02, 1.72).

**Table 3:** Well-being means on items asked only of caregivers of preschool children among 2- to 5-year-olds by hours per day of total screen time (with controls) and *d*'s comparing cells, U.S., 2016.

	None (0)	<1 h	1 h	2 h	3 h	4 h	5 h	6 h	7+ h	*d* 0 vs. 1 h	*d* 1 h vs. 4 h	*d* 1 h vs. 7+ h
Affectionate	2.98 (0.14) 321	2.95 (0.21) 754	2.96 (0.19) 1714	2.96 (0.20) 2704	2.96 (0.20) 1857	2.94 (0.26) 917	2.93 (0.27) 281	2.95 (0.23) 243	2.93 (0.34) 266	−0.11*	−0.10*	−0.14
Smiles and laughs	2.98 (0.11) 322	2.98 (0.14) 755	2.98 (0.12) 1715	2.98 (0.15) 2705	2.99 (0.12) 1858	2.98 (0.18) 919	2.96 (0.24) 280	2.97 (0.19) 246	2.98 (0.18) 266	0.00	0.00	0.00
Bounces back	2.74 (0.44) 321	2.70 (0.49) 751	2.73 (0.46) 1708	2.72 (0.48) 2701	2.72 (0.49) 1857	2.64 (0.56) 915	2.63 (0.57) 281	2.68 (0.52) 246	2.68 (0.55) 265	−0.01	−0.18*	−0.11
Does not lose temper	3.05 (0.53) 181	3.05 (0.48) 400	3.05 (0.53) 1113	3.03 (0.51) 1987	2.99 (0.53) 1406	2.96 (0.57) 709	2.89 (0.59) 205	2.82 (0.68) 190	2.89 (0.68) 210	0.00	−0.16*	−0.29*
Can calm down when excited	3.09 (0.60) 180	3.00 (0.57) 397	3.00 (0.61) 1112	3.02 (0.61) 1984	2.99 (0.62) 1403	2.98 (0.62) 710	2.85 (0.65) 205	2.81 (0.68) 191	2.86 (0.68) 210	−0.15	−0.03	−0.23*
Switch tasks without anxiety or anger	3.49 (0.56) 182	3.44 (0.54) 400	3.49 (0.55) 1114	3.48 (0.56) 1986	3.41 (0.58) 1404	3.40 (0.60) 711	3.38 (0.57) 205	3.32 (0.57) 190	3.39 (0.63) 211	0.00	−0.16*	−0.20*
Task self-control (4 items)	3.16 (0.37) 177	3.10 (0.42) 394	3.08 (0.40) 1107	3.06 (0.40) 1980	3.00 (0.42) 1396	2.98 (0.40) 704	2.96 (0.47) 201	2.86 (0.49) 189	2.91 (0.46) 207	−0.21*	−0.25*	−0.41*
Plays well with others	3.35 (0.54) 180	3.39 (0.53) 399	3.41 (0.54) 1114	3.39 (0.55) 1985	3.35 (0.55) 1407	3.35 (0.57) 711	3.27 (0.66) 203	3.34 (0.61) 190	3.40 (0.61) 210	0.11	−0.11*	−0.01
Empathy	3.24 (0.70) 182	3.32 (0.66) 399	3.32 (0.66) 1115	3.30 (0.67) 1989	3.27 (0.70) 1409	3.25 (0.70) 711	3.23 (0.74) 205	3.27 (0.68) 191	3.31 (0.71) 211	0.12	−0.10*	−0.01

Notes: 1. Within levels of screen time, numbers in each cell are: well-being means, SDs in parentheses, and *n*'s. 2. *d* = effect size corresponding to difference in standard deviations. 3. * = *t*-test comparing cells significant at *p* < .05.

There were few significant associations between screen time and social interaction items such as showing affection to caregivers, smiling and laughing, playing well with others, or showing empathy for others (see Table 3). However, several of these items (particularly showing affection and smiling and laughing) suffered from an extreme lack of variance with 95% of caregivers agreeing this was true of the child, limiting their utility.

## 4 Discussion

Children and adolescents who spent more time using screen media were lower in psychological well-being than low users. High users of screens were significantly more likely to display poor emotion regulation (not staying calm, arguing too much, being difficult to get along with), an inability to finish tasks, lower curiosity, and more difficulty making friends. Caregivers also described high users as more difficult to care for and as lower in self-control. Among adolescents, high (vs. low) users were also twice as likely to have received diagnoses of depression or anxiety or needed treatment for mental or behavioral health conditions. Moderate users were also significantly more likely than low users of screens to be low in well-being and, among 14- to 17-year-olds, to have been diagnosed with depression or anxiety or need mental health treatment. Non-users generally did not significantly differ in well-being from low users of screens.

The AAP recommendations set specific screen time limits only for children 5 years of age and younger. One set of researchers questioned these limits based on null correlations between screen time and four well-being items included in the 2011 NSCH (Przybylski and Weinstein, 2018). However, the more comprehensive set of well-being items in the 2016 NSCH produces significant associations between screen time and well-being on 18 of 19 indicators, providing substantial support for screen time limits. Notably, we found that the association between screen time and low well-being was larger for adolescents than for younger children, consistent with at least one earlier study (Rosen et al., 2014). This suggests that the AAP and other organizations focused on public health might consider extending recommendations for specific limits on screen time to preteens and teens.

It is worth speculating about why the associations between screen time and psychological well-being were larger among adolescents. One possibility is that adolescents, compared to younger children, are considerably more likely to have social media accounts and to spend more time online. Peer relationships are particularly important for adolescents (Fuligni and Eccles, 1993), and if social media replaces face-to-face interaction, that may have a negative impact on well-being and mental health. Time spent on social media, gaming, and online is more strongly correlated with low well-being than watching TV/videos, and TV/videos are the more common screen activity for younger children (Rosen et al., 2014). Unfortunately, associations with well-being for TV vs. other screen activities cannot be determined in this dataset as time spent on TV and electronic gaming was included in the same item.

Adolescents are also more likely than younger children to have their own smartphone (Rosen et al., 2014), which allows the use of technology in more situations. This may increase the possibility of Internet addiction, excessive gaming, or problematic social media use, which has been linked to low well-being (Satici and Uysal, 2015). It may also increase the impact on sleep, as smartphones may be brought into the bedroom or even the bed, with negative impacts on sleep duration and/or sleep quality (Twenge et al., 2017). Smartphones may also be used during face-to-face social interactions, which may negatively impact those interactions and blunt their usually positive impact on well-being (Dwyer et al., 2018).

Due to the cross-sectional design of the study, it is not possible to determine if screen time leads to low well-being, low well-being leads to screen time, or both. However, several longitudinal studies have found that increases in recreational screen time precede lower psychological well-being among children and adolescents (Allen and Vella, 2015; Babic et al., 2017; Hinkley et al., 2014; Kim, 2017) as well as among adults (Kross et al., 2013; Schmiedeberg and Schröder, 2017; Shakya and Christakis, 2017). In addition, experiments have shown that the presence of smartphones can lower enjoyment during social interactions (e.g., Dwyer et al., 2018; Kushlev et al., 2017) and that abstaining from social media use for one week can increase well-being (Tromholt, 2016). In other studies, the relationship appears to be reciprocal, with screen time and well-being each causing the other (Gunnell et al., 2016). These studies suggest that at least some of the causation moves from screen time to lower well-being. Regardless of the direction of causation, however, these associations have meaningful clinical implications for screening and intervention. For example, an assessment of screen time may help providers identify children and adolescents at higher risk for mental health issues and broach the topic of the possible role of screen time in mental health among these individuals.

These data are limited by several factors. First, screen time was reported by caregivers and not the children or adolescents themselves. This likely resulted in underestimates of screen time and may have unknown interactions with reports of well-being. The well-being measures may be influenced by caregivers' perceptions and may underreport issues that children do not disclose to their parents. This is likely to be less of an issue for the items on diagnoses of anxiety and depression and reports of taking medication. In addition, informant reports are often considered a strength in study design, as in some cases observers can provide more accurate information than is possible via self-report (Connelly and Ones, 2010); that is especially true for younger children. Second, the survey assessed only weekday screen time, and screen time may be higher on weekends. However, previous research found similar associations with well-being for weekday and weekend use of screen media (Przybylski and Weinstein, 2017). Weekday screen time is also likely to vary less and thus may produce a more reliable estimate. Third, the survey items combined TV and electronic games into one question, which allowed only an analysis of total screen time and not any differentiation between legacy media (TV) and digital media (electronic games, Internet, social media, etc.). Fourth, although the Census Bureau attempted to recruit a representative sample, the response rate was not 100% and some groups (such as Black Americans) are under-represented relative to their percentage of the total U.S. population in the final sample.

In summary, these results show a negative association between screen time and psychological well-being among children and adolescents. Across a diverse array of well-being measures, including measures of self-control, relationships with caregivers, emotional stability, diagnoses of anxiety and depression, and mental health treatment, psychological well-being was progressively lower from 1 h a day of screen time to 7 or more hours a day of screen time, particularly among adolescents. The significant association between screen time and well-being may have important clinical implications for the mental and physical health of children and adolescents and for developing guidelines for specific screen time limits for older children and adolescents.

## Funding

This study received no funding.

## Conflict of interest statement

The authors declare that there are no conflicts of interest.

## Appendix A Supplementary data

Supplementary material Image 1
