# Supplementary material for: Associations between screen time and lower psychological well-being among children and adolescents: Evidence from a population-based study
Source: Prev Med Rep. 2018 Oct 18;12:271–83. doi: 10.1016/j.pmedr.2018.10.003 (PMC6214874; doi:10.1016/j.pmedr.2018.10.003)
Supplement: Supplementary file 1 — Supplementary material [file mmc1.docx]

Supplementary Material

The well-being items included:

1. *Easy child* (3 items). “During the past month, how often have you felt: that [child’s name] is much harder to care for than most children his or her age; that [child’s name] does things that really bother you a lot; angry with [child’s name]?” Response choices were on a 5-point scale from never to always, with responses reverse scored (alpha = .79).

2. *Curious*. “[Child’s name] shows interest and curiosity in learning new things.” Response choices were on a 3-point scale from definitely true to not true.

3. *No difficulty making friends*. “Compared to other children his or her age, how much difficulty does [child’s name] have making or keeping friends?” 3-point scale from no difficulty to a lot of difficulty. Reverse scored.

4. *Calm when challenged*. “[Child’s name] stays calm and in control when faced with a challenge.” 3-point scale from definitely true to not true.

5. *Finishes tasks started*. “[Child’s name] works to finish tasks he or she starts.” 3-point scale from definitely true to not true.

6. *Does not argue too much*. “[Child’s name] argues too much.” 3-point scale from definitely true to not true. Reverse scored.

7. *Anxiety*. “Has a doctor or other health care provider ever told you that [child’s name] has anxiety problems?” Response choices were yes or no. An additional question asked if the anxiety was mild, moderate, or severe (coded 1-3).

8. *Depression*. “Has a doctor or other health care provider ever told you that [child’s name] has depression?” Response choices were yes or no. An additional question asked if the depression was mild, moderate, or severe (coded 1-3).

9. *Treated or needed to be treated by mental health professional*. “During the past 12 months, has [child’s name] received any treatment or counseling from a mental health professional? Mental health professionals include psychiatrists, psychologists, psychiatric nurses, and clinical social workers.” Response choices were “yes,” “no, but this child needed to see a mental health professional,” and “no, this child did not need to see a mental health professional.” We recoded the first two responses as affirmative.

10. *Took medication for psychological issue*. “During the past 12 months, has [child’s name] taken any medication because of difficulties with his or her emotions, concentration, or behavior?” Response choices were yes or no.

Some well-being items were only asked of caregivers of children 5 or under:

11. *Affectionate*. “[Child’s name] is affectionate and tender with you.” 3-point scale from definitely true to not true.

12. *Smiles and laughs*. “[Child’s name] smiles and laughs a lot.” 3-point scale from definitely true to not true.

13. *Bounces back*. “[Child’s name] bounces back quickly when things do not go his or her way.” 3-point scale from definitely true to not true.

14. *Does not lose temper*. “How often does [child’s name] lose control of his or her temper when things do not go his or her way?” 4-point scale from none of the time to all of the time. Reverse scored.

15. *Can calm down*. “How often does [child’s name] calm down when excited or all wound up?” 4-point scale from none of the time to all of the time. Reverse scored.

16. *Not angry or anxious when switching tasks*. “How often does [child’s name] become angry or anxious when going from one activity to another?” 4-point scale from none of the time to all of the time. Reverse scored.

17. *Task self-control* (4 items). “How often is [child’s name] easily distracted?” “How often does [child’s name keep working at something until he or she is finished?” “When he or she is paying attention, how often can [child’s name] follow instructions to complete a simple task?” “Compared to other children his or her age, how often is [child’s name] able to sit still?” 4-point scale from none of the time to all of the time. (alpha = .65).

18. *Plays well with others*. “How often does [child’s name] play well with others?” 4-point scale from none of the time to all of the time.

19. *Empathy*. “How often does [child’s name] show concern when others are hurt or unhappy?” 4-point scale from none of the time to all of the time.
